# Love-Mode MEMS Devices for Sensing Applications in Liquids

**DOI:** 10.3390/mi7010015

**Published:** 2016-01-21

**Authors:** Cinzia Caliendo, Smail Sait, Fouad Boubenider

**Affiliations:** 1 Istituto di Acustica e Sensoristica “O.M. Corbino”, IDASC, Consiglio Nazionale delle Ricerche, CNR, Via del Fosso del Cavaliere 100, 00133 Rome, Italy; 2 Laboratory of Physics of Materials, Team “Waves and Acoustic”, University of Sciences and Technology, Houari Boumedienne, B.P. 32 El Allia, Bab-Ezzouar, 16111 Algiers, Algeria; saitsmail@yahoo.fr (S.S.); fboubenider@yahoo.fr (F.B.); 3 Faculté des Sciences, Université M. Mammeri, BP 17 R.P., 15000 Tizi-Ouzou, Algeria

**Keywords:** microsensors, acoustic modes, piezoelectric materials

## Abstract

Love-wave-based MEMS devices are theoretically investigated in their potential role as a promising technological platform for the development of acoustic-wave-based sensors for liquid environments. Both single- and bi-layered structures have been investigated and the velocity dispersion curves were calculated for different layer thicknesses, crystallographic orientations, material types and electrical boundary conditions. High velocity materials have been investigated too, enabling device miniaturization, power consumption reduction and integration with the conditioning electronic circuits. The electroacoustic coupling coefficient dispersion curves of the first four Love modes are calculated for four dispersive coupling configurations based on a *c*-axis tilted ZnO layer on wz-BN substrate. The gravimetric sensitivity of four Love modes travelling at a common velocity of 9318 m/s along different layer thicknesses, and of three Love modes travelling at different velocity along a fixed ZnO layer thickness, are calculated in order to design enhanced-performance sensors. The phase velocity shift and attenuation due to the presence of a viscous liquid contacting the device surface are calculated for different thicknesses of a *c*-axis inclined ZnO layer onto BN half-space.

## 1. Introduction

Chemical sensors based on the propagation of surface acoustic waves (SAWs) are usually based on a delay line or resonator configuration with the acoustic wave path covered by a membrane sensible to a specific target analyte. When the specific target molecule interacts with the membrane, such as antibody and microorganism, the membrane changes its mechanical properties, thus affecting the phase velocity and/or propagation loss of the wave. As a consequence, the device resonance frequency and/or attenuation changes can be used to quantify the sensors response. The performances of the electroacoustic sensors are affected by the behaviour of the thin membrane as well as by the device design. The membrane is required to be reversible, repeatable, stable in time and selective. The electroacoustic device to be used for sensing applications in liquid environment must involve the propagation of in-plane polarized modes since these modes do not radiate energy into the liquid. In this context, Love wave surface modes are promising platforms for biosensing applications. The Love wave is a shear horizontally (SH) polarized wave that propagates on the surface of a piezoelectric half-space or of a thin piezoelectric layer deposited on a semi-infinite substrate. The SH polarization ensures a weak acoustic energy loss in the liquid phase contacting the device surface, while the latter ensures a strong energy confinement in the piezoelectric layer. Love wave sensors have been attracting the interest of many researchers since the early 1990s [1,2,3] due to their remarkable features: the lower acoustic wave velocity of the layer results in the acoustic wave being guided through the layer itself, resulting in minimal acoustic losses into the bulk of the substrate or into the liquid contacting the sensor surface. Moreover, improved sensor performances can be achieved by choosing a proper layer thickness, which increases the sensitivity of the device towards changes in physical properties at its surface, including mass loading.

In this study, we theoretically studied the propagation of Love wave along the surface of some piezoelectric substrates (AlN, InN, ZnO, GaN, ST quartz): the phase velocity and the electroacoustic coupling coefficient (*K*^2^) were calculated for different *c*-axis tilt angles with respect to the surface normal. In order to increase the Love wave velocity and *K*^2^ with respect to those obtained for single material substrates, we investigated a dispersive structure consisting in a thin piezoelectric ZnO layer on top of the surface of a semi-infinite BN substrate. The ZnO layer has its *c*-axis inclined onto the surface while the BN has its *c*-axis normal to the free surface. The *K*^2^ and velocity dispersion curves, and the gravimetric sensitivities were calculated for different Love modes, in order to compare the overall behavior of the sensors based on different Love modes. Finally, the first Love mode attenuation and relative velocity shift due to the presence of a liquid (water) contacting the device surface were calculated for different ZnO layer thicknesses, thus confirming that the sensor behavior can be enhanced by proper choice of material parameters (*i.e*., types and thicknesses) of the layered waveguide structure.

## 2. Love Wave Propagating Along a Piezoelectric Half-Space

**Figure 1:**
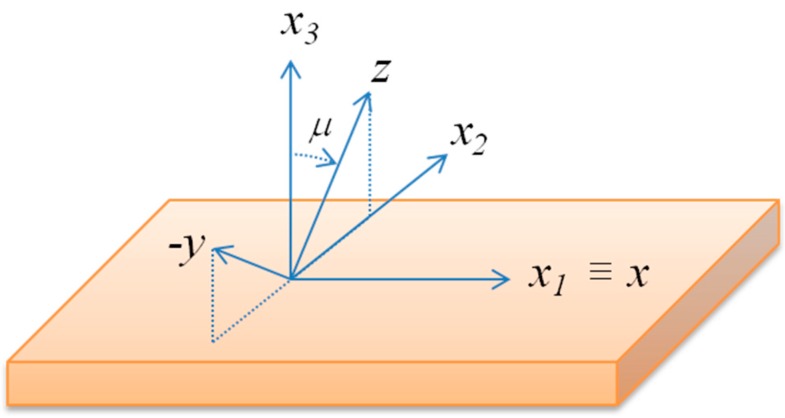
The laboratory coordinate system (*x_1_*, *x_2_* and *x_3_*) and the propagating medium crystallographic axis (*x*, −*y* and *z* ≡ *c*); μ is the *c*-axis tilt angle respect to *x_3_*.

The propagation of the Love wave can be excited and detected by means of interdigitated transducers (IDTs), as well as for the SAWs. Referring to a coordinate system with *x*_1_ and *x*_3_ parallel to the wave propagation direction and normal to the substrate surface, respectively, the electrically coupled Love wave requires the piezoelectric constants *e*_16_ and, or *e*_36_ to be different from zero. A (0002) oriented piezoelectric material of the hexagonal system (class 6 mm) has no piezoelectric coefficients that couple to shear deformation, hence the excitation of shear waves can be obtained by tilting the *c*-axis orientation by an angle μ from the normal, as shown in Figure 1.

**Figure 2:**
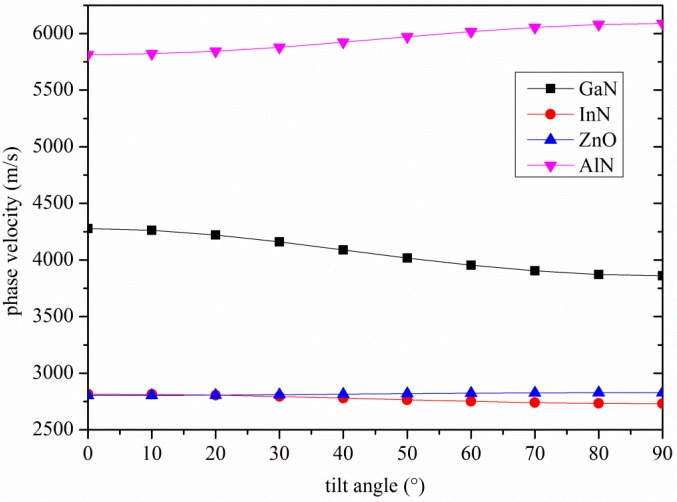
The phase velocity of the Love wave travelling along the *x* propagation direction of AlN, GaN, InN and ZnO substrates *vs.* the *c*-axis tilt angle.

**Figure 3:**
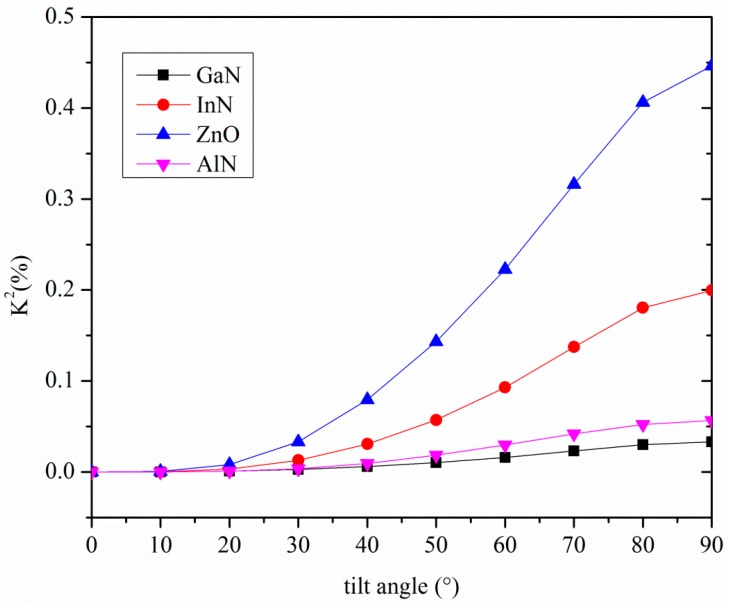
The *K*^2^ of the Love wave travelling along the *x* propagation direction of AlN, GaN, InN and ZnO substrates *vs.* the *c*-axis tilt angle.

As a result, the electric field is coupled only with the particle motion in the shear horizontal (SH) direction, *U*_2_. *U*_2_ is maximum at the free surface of the substrate and remains constant for about one wavelength in depth, and then it decreases asymptotically. Therefore the electroacoustic coupling coefficient is affected by the *c*-axis inclination angle from the vertical as well as by the wave propagation direction. The phase velocity and *K*^2^ of the Love wave travelling along the surface of some piezoelectric substrates were calculated by using a Matlab routine in the lossless approximation; the material data (mass density, elastic, piezoelectric, and dielectric constants) are referred to in [4,5]. By tilting the *c*-axis of the AlN, InN, ZnO and GaN piezoelectric half-spaces with respect to the surface normal, the phase velocity of the Love wave changes, as well as the coupling coefficient, as shown in Figure 2 and Figure 3.

**Figure 4:**
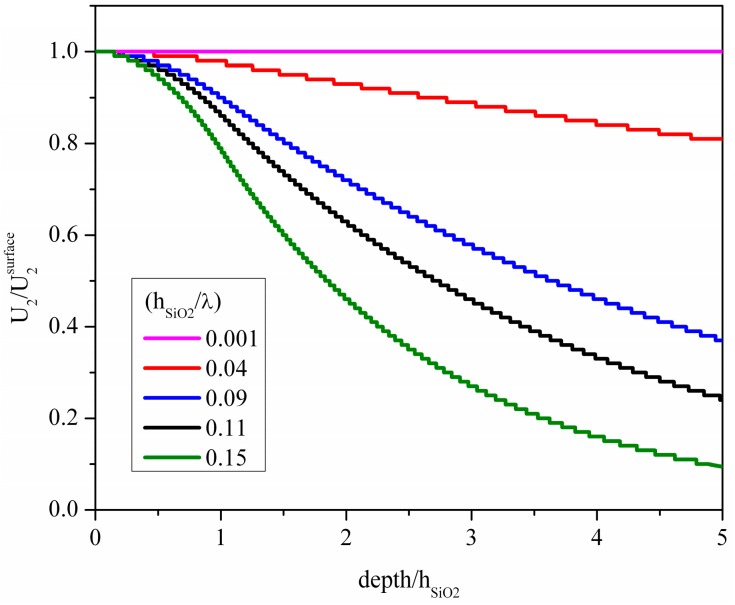
The normalized particle displacement component of the Love wave *vs*. the acoustic wave penetration depth normalized to the SiO_2_ layer thickness *h*_SiO2_; the SiO_2_ thickness to wavelength ratio, *h*_SiO2_/λ, is the running parameter.

A piezoelectric material widely used for the implementation of Love wave based sensors [6,7,8,9] is the ST-Y quartz where the wave propagation direction is orthogonal to the crystalline *x* axis direction in order to allow the excitation of purely shear polarized modes by means of the IDTs: 4991 m/s is the wave velocity and 0.018% is the *K*^2^. The weak piezoelectric coupling of this material can be improved by covering the free surface of the quartz substrate with a thin SiO_2_ layer. This layer plays the twofold role of increasing the electroacoustic coupling coefficient, while trapping the acoustic energy near the surface of the substrate. Figure 4 shows the particle displacement component normalized to its surface value, *U*_2_/*U*_2_^surface^, *vs.* the depth of the SiO_2_/quartz propagating medium; the running parameter is the SiO_2_ film thickness normalized to the acoustic wavelength (*h*_SiO2_/λ). The abscissa of the graph has been normalized to *h*_SiO2_ to make visible how the layer thickness affects the acoustic field at the layer/substrate interface (depth/*h*_SiO2_ equal to 1) and inside the substrate. With increasing SiO_2_ layer thickness, more and more energy is trapped inside the layer and, for *h*_SiO2_/λ > 0.07, the displacement component value at the quartz surface starts becoming ever lower, while it remains equal to 1 at the layer free surface. As described in [7,10], to cite just a few, the SiO_2_ layer thickness affects the mass sensitivity of the sensor to the viscous liquid characteristics, as well as the insertion loss, temperature coefficient of oscillation frequency and frequency noise.

**Figure 5:**
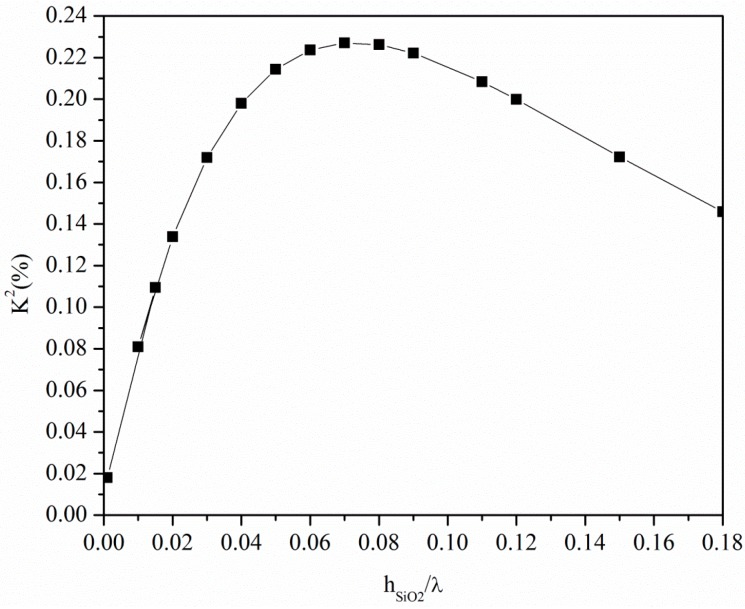
The *K*^2^ of the ST-Y quartz *vs*. the SiO_2_ normalized thickness.

The presence of the SiO_2_ layer affects the acoustic field distribution as well as the *K*^2^ of the SiO_2_/ST-quartz substrate, as shown in Figure 5. The *K*^2^ improves with increasing layer thickness up to the threshold value *h*_SiO2_/λ = 0.07, while it decreases for higher layer thicknesses. The velocity as well as the *K*^2^ values of the SiO_2_/quartz substrate are much lower than those referred to the *c*-axis inclined AlN, while only the *K*^2^ of the GaN, InN and ZnO bare substrates is competitive with that of the SiO_2_/quartz substrate. Among these materials, AlN is the fastest while ZnO ensures the highest *K*^2^ value. Moreover, these piezoelectric materials can be grown in thick film form onto non-piezoelectric substrates, such as silicon, and, provided that their thickness is greater than the acoustic wavelength, are suitable for the implementation of Love-wave-based sensor platforms for applications in liquid environments.

## 3. Love Modes Propagating Along a Piezoelectric Layer onto a Non Piezoelectric Half-Space

**Figure 6:**
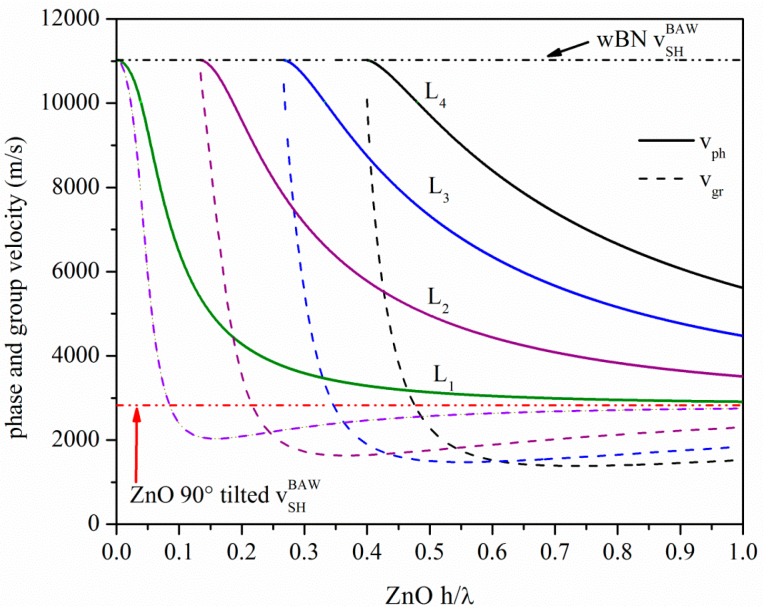
The phase and group velocity dispersion curves of the Love modes travelling along the *x* propagation direction of *c*-axis inclined ZnO/wz-BN.

Dispersive Love wave devices can also be developed which consist of a thin wave guide layer on top of the surface of a substrate. The essential condition for the propagation of the Love wave along a layered medium is that the shear bulk wave velocity (SHBAW) in the layer is smaller than that in the substrate, *i.e.*, the layer loads the substrate. As a result, multiple Love modes propagate which involve only displacements perpendicular to the sagittal plane. The phase velocity *v_ph_* of the first mode becomes equal to the SHBAW velocity of the substrate with decreasing layer thickness, while it asymptotically reaches the SHBAW velocity of the layer with increasing layer thickness. The higher order Love modes have a cut off when their *v_ph_* equals the SHBAW velocity of the substrate and at the cut off the group velocity *v_gr_* equals *v_ph_*. Unlike the *v_ph_*, the *v_gr_* dispersion curve of each mode has infinite slope. *v_ph_* and *v_gr_* approach asymptotically the SHBAW velocity of the layer with increasing the layer thickness, as illustrated by the dispersion curves in Figure 6 for a *c*-axis inclined ZnO film on a semi-infinite wurtzite BN substrate. The *v_gr_* was calculated from the *v_ph_* by the following formula: vgr=vph[1+hλvphdvphd(hλ)], being λ the acoustic wavelength and *h* the layer thickness. In Figure 6, the velocities of the SHBAW of the wz-BN and of the *c*-axis inclined ZnO are shown as well. Many authors have demonstrated the feasibility of the growth of ZnO layers with an in-plane *c*-axis onto different substrates, such as on R plane sapphire [11,12], onto Au/SiO_2_ substrate [13], onto Au [14] on an indium tin oxide (ITO) on quartz substrate [15], and on Al polycrystalline films [16], to name just a few. wz-BN films are currently grown by sputtering (DC or RF) and by hollow cathode arc evaporation apparatus on Si(001) and Si(111), respectively and by chemical vapor deposition: at the present, the growth of BN presents a viable route towards large-scale manufacturing of BN substrates [17,18,19,20]. Thus, the feasibility of the proposed ZnO *c*-axis inclined/wz-BN acoustic waveguide is reliable and compatible with semiconductor processing techniques, provided that the BN thickness is greater than the acoustic wavelength. Such multilayer structure offers the advantage of providing the monolithic integration of the device with the signal processing electronics. Other combinations of materials can be explored also to improve the thermal stability (by choosing materials with temperature coefficient delay opposite in sign) [21] and resistance to harsh environments (choosing materials able to survive at high temperatures and in chemically aggressive environments).

**Figure 7:**
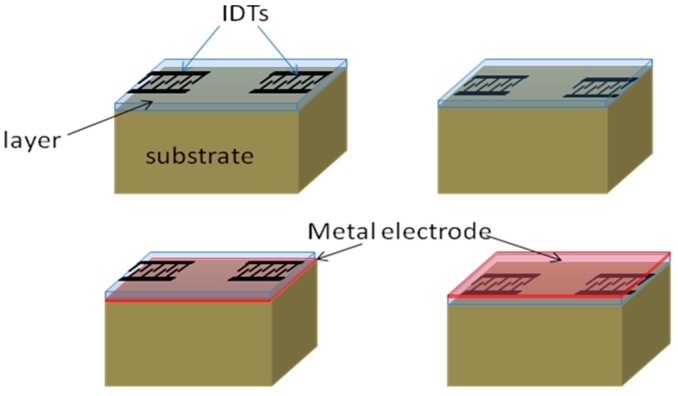
The four different coupling configurations.

In designing a Love wave device, an important feature to be obtained is low insertion loss, which can be achieved by selecting a material with a large *K*^2^. The value of *K*^2^ is directly related to the IDT electrical-to-mechanical energy conversion efficiency; hence, it determines the radiation resistance of the transducer that is fabricated on the substrate and the piezoelectric guiding layer. The layer/substrate combination allows the implementation of four different coupling configurations, shown in Figure 7, with the IDTs placed on one of the ZnO layer surfaces, with or without a floating electrode on the opposite one. The configuration called substrate/film/transducer (SFT) refers to a coupling structure with the IDTs positioned on the ZnO free surface: when a floating metallic plane (M, metal) is placed at the ZnO/BN interface, the configuration is called SMFT. The configuration called substrate/transducer/film (STF) refers to a coupling structure with the IDTs positioned at the ZnO/BN interface: when the metallic plane is positioned at the ZnO free surface, the configuration is called STFM.

For SAWs the *K*^2^ can be defined in terms of the piezoelectric coefficient, elastic constants and dielectric permittivity, *K*^2^ = *e*^2^/*c*ε, being the tensor subscripts dropped: the appropriate values of these constants depend on the crystallographic orientation of the piezoelectric material [22]. A simpler method for calculating the efficiency of excitation of acoustic surface waves by means of interdigital transducers [23] is based on the calculation of the wave velocity change due to a change in the electric field boundary conditions. When a thin, massless, perfectly conducting metal film is deposited on the surface of a piezoelectric layer, the potential and the longitudinal electric field at the piezoelectric surface layer are zero, and the wave velocity is reduced by an amount which can be regarded as a measure for the coupling strength between the wave and the metal surface electrode. The higher the velocity change, the higher the coupling to an electrode grating transducer which responds mainly to the tangential field. For piezoelectric materials with a weak piezoelectric coupling coefficient, this quantity can be written to a good approximation [24] as *K*^2^ ≈ 2·Δ*v*/*v_f_* = 2·(*v_f_* − *v_m_*)/*v_f_*, where *v_m_* and *v_f_* are the velocities calculated in the metalized and free ZnO boundary condition. For the layered structures depicted in Figure 7, the IDTs and the metal plane can be positioned onto one of the two surfaces of the layer, thus the effects of four different electrical boundary conditions at the ZnO interfaces must be considered in the calculation of the phase velocity and hence of the *K*^2^. By denoting as *v_ij_* (for *i, j* = *m, f*) the wave velocity referred to the electrical boundary conditions of the lower and upper layer surface, the following approximated formulas were used to calculate the coupling constant of the four structures:

KSFT2=2·[(vff−vfm)vff]

KSTF2=2·[(vff−vmf)vff]

KSMFT2=2·[(vmf−vmm)vmf]

KSTFM2=2·[(vfm−vmm)vfm]

**Figure 8:**
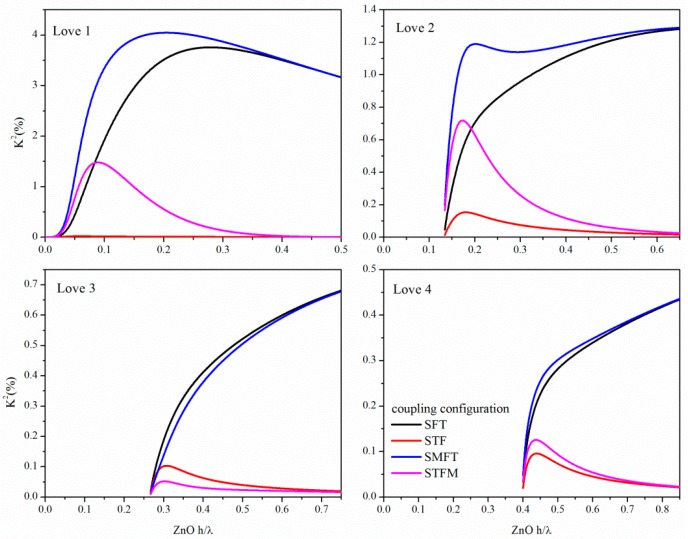
The *K^2^* dispersion curves of the four coupling configurations calculated for the first four Love modes propagating along 90° tilted ZnO film onto BN substrate.

The *K^2^* dispersion curves were theoretically calculated for the first four Love modes propagating in the four different coupling structures, as shown in Figure 8. The mechanical effect of the metallization was ignored as the metallization is assumed to be infinitely thin. As can be seen from Figure 8, the Love modes propagating along the dispersive structure are highly sensitive to the electrical boundary conditions.

With increasing the Love mode order, ever decreasing *K^2^* values can be reached by the four coupling configurations: the SMFT of the first mode reaches the highest *K^2^* value since the metallization on the ZnO side opposite the IDTs strongly enhances the vertical electric field in the ZnO layer.

**Figure 9:**
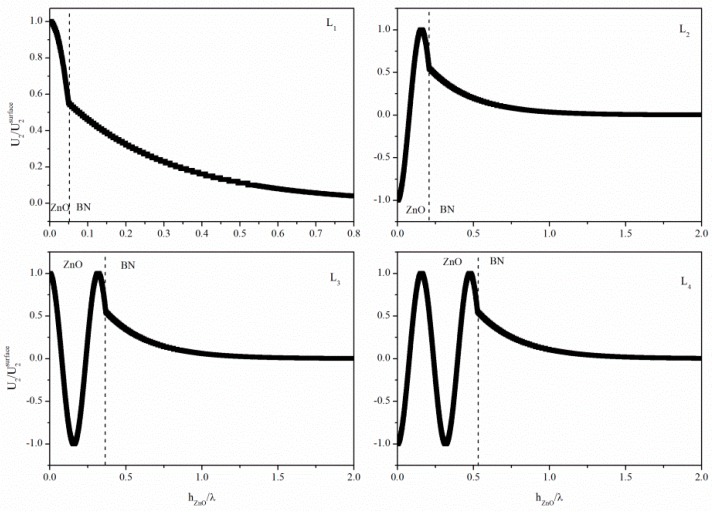
The particle displacement component of the first four Love modes travelling at a common velocity of 9318 m/s along different ZnO layer thicknesses (*h*/λ = 0.050, 0.209, 0.369 and 0.528, respectively).

Figure 9 shows the displacement component profile for the first four Love modes travelling at a common velocity of 9318 m/s along different ZnO layer thicknesses (*h*/λ = 0.050, 0.209, 0.369 and 0.528, respectively). As can be seen, the displacement in the ZnO layer varies sinusoidally while the displacement in the BN substrate has a simple exponential decay with depth below the interface for all the modes. The slope of the curve of the displacement amplitude *vs*. depth is zero at the free interface for all Love modes. Every Love mode is suitable for liquid sensing applications as the particle displacement component is at the maximum at the free surface that contacts the liquid.

## 4. Love-Mode Sensors

### 4.1. Gravimetric Sensitivity

The gravimetric sensitivity *S* of the Love waves sensor was theoretically estimated by calculating the velocity change that the modes undergo when the free ZnO layer surface is covered by a mass *m* = ρ*_m_·h_m_*, being ρ*_m_* and *h_m_* the added mass density and thickness. The *S* of the Love modes travelling at equal-velocity (*v* = 9318 m/s) along different ZnO film thickness values was calculated as *S* = (Δ*v*/*v*)/*m*, with Δ*v = v’− v*, *v* and *v’* the mode velocity along the mass-covered and bare surface. Table 1 lists the mode order, the layer thickness, the theoretically estimated *S* and the slope of the phase velocity dispersion curve, dvphd(h/λ), evaluated at the corresponding ZnO layer thickness. With increasing mode order, *S* drastically decreases and the linear behavior of the relative velocity change *vs.* the added mass is restricted to smaller *h_m_* values, as shown in Table 1. The highest *S* value correspond to the largest slope value; the decreasing values of sensitivity correspond to decreasing values of the slope. This fact confirm that the optimized sensor sensitivity corresponds to strong velocity dispersion.

The gravimetric sensitivity of the Love modes travelling along equal ZnO layer normalized thickness (*h*/λ = 0.3) was theoretically predicted and the calculated sensitivities are listed in Table 2, together with the mode order, the corresponding *v_ph_* and the *v_ph_* dispersion slope. As already observed for the data listed in Table 1, the highest values of sensitivity correspond to the largest value of the velocity dispersion slope.

**Table 1:** Love mode orders, ZnO normalized thickness, gravimetric sensitivity values and the phase velocity slope: the data refer to modes travelling at the same velocity (9318 m/s).

Love mode order	ZnO *h*/λ	S (m^2^·kg^−1^·λ^−1^) *	*v_ph_* slope
L_1_	0.0501	−13 × 10^−4^	−67,118
L_2_	0.2093	−5.7 × 10^−4^	−29,635
L_3_	0.3689	−3.7 × 10^−4^	−18,993
L_4_	0.5280	−2.7 × 10^−4^	−13,996

Note: * The *S* was calculated for an added mass layer thickness *h_m_*/λ ranging from 0 to 0.04.

**Table 2:** Love mode orders, phase velocity, gravimetric sensitivity values *S* and the phase velocity slope: the data are referred to Love modes travelling at different velocities along the same ZnO layer thickness (*h*/λ = 0.3).

Love mode	Velocity (m/s)	S (m^2^·kg^−1^·λ^−1^) *	*v_ph_* slope
L_1_	1,0653.4316	−1.3 × 10^−4^	−17,080
L_2_	7,155.6581	−4.2 × 10^−4^	−18,087
L_3_	3,587.5888	−3.3 × 10^−4^	−4,267

Note: * The *S* was calculated for an added mass layer thickness *h_m_*/λ ranging from 0 to 0.04.

The gravimetric sensitivity slightly increases with increasing the mode order and the linear behavior of Δ*v*/*v* *vs.* the added mass layer thickness is restricted to smaller ranges. By comparing the data shown in Table 1 and Table 2, it appears clear that the sensitivity is affected by both the ZnO thickness and the phase velocity value.

### 4.2. Viscosity Sensitivity

**Figure 10:**
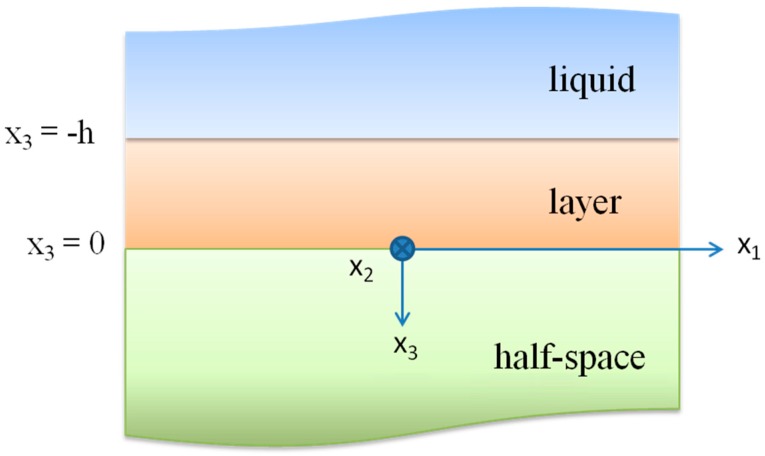
The geometry of the Love wave waveguide. The layer upper surface (*x*_3_ = −*h*) is in contact with the viscous liquid.

A shear horizontally polarized surface wave couples with the viscosity of the adjacent liquid because its displacement is transverse to the propagation direction and parallel to the substrate surface. Most of the wave energy is localized near the surface, within a depth of about one wavelength. Assuming a non-slip boundary condition at the layer surface, a thin film of liquid becomes entrained with the shear movement. The characteristic decay length of this entrainment is δ=2ηρω, *i.e.*, the penetration depth of the wave inside the liquid, where η and ρ are the viscosity and the mass density of the liquid, and ω = 2π*f* stands for the angular frequency. The Love wave propagation is affected by the viscous loading in two ways: the mass loading of the waveguide from the viscously entrained liquid of thickness δ, which yields a change in the wave number of the wave, and the viscous losses in the liquid, that damp the wave. The dispersion curves of the Love modes propagating along the *c*-axis inclined ZnO/isotropic BN medium were theoretically calculated in the ZnO and BN lossless approximation. The mechanical displacement component of the Love wave *U*_2_ is polarized along the *x*_2_ axis, perpendicular to the direction of propagation *x*_1_. The waveguide surface is at *x*_3_ = −*h*, being h the layer thickness. We considered a two-dimensional problem with no variation along the *x*_2_ axis and restricted the following calculations to the propagation of the first Love mode. The geometry of the Love wave layered structure is shown in Figure 10: it involves an isotropic elastic substrate and layer; the top surface of the layer is loaded by a viscous Newtonian liquid.

A Love wave solution occurs when the substrate thickness can be considered infinite, the shear velocity of the substrate is greater than the shear velocity of the layer, and the wave displacement U_2_ decays with depth in the liquid as well in the substrate. The layer and substrate are assumed to behave as ideal linear elastic materials: the mechanical displacement field U_2_ in the layer (0 < *x*_3_ < −*h*) and in the substrate (*x*_3_ > 0) satisfies the equations of motion as follows:

(1a) $$\frac{1}{v_{2}^{2}}\frac{\partial^{2}u_{2}^{film}}{\partial t^{2}}=\left(\frac{\partial^{2}}{\partial x_{1}^{2}}+\frac{\partial^{2}}{\partial x_{3}^{2}}\right)u_{2}^{film}\;\text{for}\;0<x_{3}<-h$$

(1b) $$\frac{1}{v_{1}^{2}}\frac{\partial^{2}u_{2}^{substrate}}{\partial t^{2}}=\left(\frac{\partial^{2}}{\partial x_{1}^{2}}+\frac{\partial^{2}}{\partial x_{3}^{2}}\right)u_{2}^{substrate}\;\text{for}\;x_{3}>0$$

which include the relevant materials parameters *v*_1_ (the shear bulk wave velocity of BN) and *v*_2_ (the shear bulk wave velocity in the ZnO layer). The liquid is considered to be a Newtonian fluid obeying the Navier–Stokes equation:

(1c) $$\frac{\partial v_{2}}{\partial t}=\frac{\eta }{\rho_{l}}\left(\frac{\partial^{2}}{\partial x_{1}^{2}}+\frac{\partial^{2}}{\partial x_{3}^{2}}\right)v_{2}\;\text{for}\;x_{3}<-h$$

where ρ*_l_* and η are the liquid density and viscosity.

The three functions describing the mechanical displacement field *U_2_* of the Love wave in the surface layer and in the substrate and the velocity field in the liquid are the following:

(2a) $$u_{2}^{film}=\left[A\cdot cos\left(qx_{3}\right)+B\cdot \sin\left(qx_{3}\right)\right]\cdot e^{j\left(kx_{1}-\omega t\right)}$$

(2b) $$u_{2}^{substrate}=\left[C\cdot e^{-bx_{3}}\right]\cdot e^{j\left(kx_{1}-\omega t\right)}$$

(2c) $$v_{2}^{liquid}=\left[D\cdot e^{-px_{3}}\right]\cdot e^{j\left(kx_{1}-\omega t\right)}$$

where *A*, *B*, *C* and *D* are constants to be calculated by the boundary conditions, ω is the angular frequency, *k* = *k*_0_ + *j*α is the complex wave number, *k*_0_ = ω/*v*_0_, *v*_0_ is the wave velocity, α is the wave attenuation, and t is time. After Equation (2a–c) has been substituted into Equation (1a–c), the wave vectors *q*, *b* and *p* can be obtained:

$$q=\sqrt{k_{1\;}^{2}-k^{2}}$$

$$b=\sqrt{k_{\;}^{2}-k_{2}^{2}}\;$$

$$p=\sqrt{k_{0\;}^{2}-j\omega \rho_{l}/\eta }$$

being $k_{1}=\frac{\omega }{v_{1}}$ and $k_{2}=\frac{\omega }{v_{2}}$. Equation (2a–c) is connected by the non-slip boundary conditions: at the interface between the layer and the substrate (*x*_3_ = 0) and at the interface between the free surface of the layer and the liquid (*x*_3_ = −*h*), the mechanical displacement and the shear stress *T*_23_ have to fulfil the conditions of continuity. By applying the boundary conditions, a system of four non linear equations for coefficients A, B, C and D are derived, being the phase velocity and attenuation α the two unknowns, while the layer thickness is fixed to the value *h* = 2 µm, and the operating frequency *f* is calculated by f=vairλ, being $v^{air}$ the Love mode velocity previously calculated by solving the motion equations relative to the layered waveguide in air. For a nontrivial solution, the determinant of this set of equations has to equal zero, and the following analytical expression for the complex dispersion equation of the Love wave is obtained [25]:

(3) $$\sin\left(qh\right)[\rho_{1}^{2}v_{1}^{4}q^{2}+\rho_{2}v_{2}^{2}bpj\omega \eta ]-\cos\left(qh\right)\left[\rho_{1}\rho_{2}v_{1}^{2}v_{2}^{2}bq-\rho_{1}v_{1}^{2}qpj\omega \eta \right]=0$$

**Figure 11:**
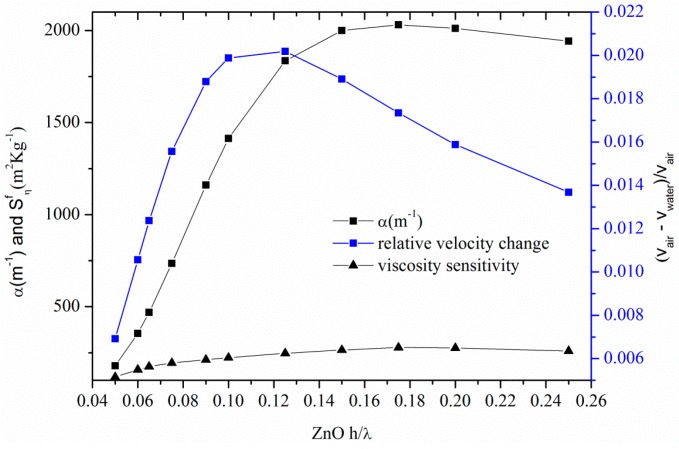
The attenuation α and the relative velocity changes due to the presence of the viscous liquids.

After separating the real and imaginary parts of Equation (3), a system of two nonlinear algebraic equations was obtained: the layer thickness (fixed at 2 µm), and both the ZnO and BN bulk shear wave velocities and density, the liquid density and viscosity were the equations parameters, while the wave velocity and attenuation were the unknowns. The system was solved numerically using the Newton-Raphson method implemented with Matlab, assuming that the free ZnO layer surface is loaded with a viscous Newtonian liquid (water) with mass density ρ_l_ = 1000 kg/m^3^ and viscosity η = 0.001 Pa·s. The perturbed operating frequency was estimated by the relation *f = v_water_/λ* where *v* is the Love wave velocity calculated by the dispersion Equation (3) and λ is the wavelength (equal to the IDTs periodicity). Figure 11 shows the attenuation α and the relative velocity change [(vair−vwater)vair] due to the presence of the viscous liquid, for different ZnO normalized thicknesses. Also shown in Figure 11 is the viscosity sensitivity, evaluated as Sηf=(vgrvph)(Δv/v)(ρlδ), *i.e.*, the relative frequency change per unit viscous liquid mass ml=ρl·δ=2ηρω, for different layer thicknesses. As it can be seen in Figure 11, there is an optimum ZnO layer thickness corresponding to the enhanced sensor sensitivity. The highest sensitivity value, equal to 280 m^2^·kg^−1^, and corresponding to an operation frequency of 354 MHz, is quite larger than that (approxmately 45 m^2^·kg^−1^) calculated in [26] and referred to a quartz substrate covered by a SiO_2_ layer, 6.5 μm thick, and operating at 124 MHz.

## 5. Conclusions

Love sensors are likely to see increased competition from devices such as Lamb wave sensors for gravimetric applications in liquid media; the acoustic plate modes have the disadvantage of requiring bulk micromachining, whereas the formers can be fabricated using well established surface micromachining. Additionally, Love wave devices are more robust, can operate in a harsh environment and have operation capability on a wireless platform, which makes it possible to operate these devices from a remote location. There is a wide range of combinations of materials of which the devices can be made to improve the velocity (choosing fast materials), the electroacoustic coupling efficiency (choosing materials with a high piezoelectric coupling), the thermal stability (by choosing materials with a temperature coefficient delay opposite in sign) and resistance to harsh environments (choosing materials able to survive at high temperatures and in chemically aggressive environments). A proper sensor device design is required to trade off mass sensitivity as well as electromechanical coupling, which affects device size and insertion loss. Since there is no material combination which outperforms all others in these two areas, the research for substrate and layer configurations with favourable properties is still in progress. In the present paper we studied conventional piezoelectric substrates, such as ST-Y quartz, and unconventional piezoelectric substrates, such as *c*-axis tilted AlN, ZnO, GaN and InN, to be used as Love wave propagating media suitable for the implementation of high-frequency, enhanced-coupling sensor devices. Our theoretical study demonstrates that Love mode devices on *c*-axis inclined ZnO/BN substrate can offer remarkable performance, with qualities such as high velocity (and hence operating frequency), quite good *K*^2^ (4%), and high sensitivity. In terms of feasibility, this acoustic waveguide is reliable and compatible with semiconductor processing techniques, thus offering the advantage of providing the monolithic integration of the device with the signal processing electronics.
